# Some American Hospitals

**Published:** 1892-01-09

**Authors:** 


### SOME AMERICAN HOSPITALS.

BY OUR SPECIAL COMMISSIONER.

### II.—SAN FRANCISCO.

THE hospitals of San Francisco consist of the United States Marine Hospital, on the Presidio Reservation ; the New City Hospital ; St. Mary's Hospital (Roman Catholic) ; State Women's Hospital, and a Hospital for Women and Children, the latter being managed almost exclusively by women, the medical staff being all women with the exception of the Orthopædic Surgeon.

It would be wearisome to give an account of each of these institutions. We will therefore content ourselves by describing the two first.

The United States Marine Hospital is built on the barrack system, and contains from sixty to eighty beds. As its name indicates, it is one of a great number of marine hospitals established and supported by the United States Government for the relief and succour of seamen employed in the Mercantile Marine.

The hospitals are served by a staff of physicians and surgeons, and employés who are engaged by the Government, constituting a separate service, and promoted mainly by seniority. Most of the hospitals on this system have been erected on the barrack principle, though here and there a large permanent building exists to which sailors are sent. In addition to the hospital proper, the Marine Hospital at San Francisco at present consists of the residences of the Surgeon-Superintendent and staff, together with an ambulance station, a store, and other necessary buildings, the institution being entirely self-contained. Water is supplied from a separate spring, and pumped up specially for the use of the hospital. The institution makes its own gas, grows its own vegetables and fruit, and utilises its own sewage for agricultural purposes.

Although the wards are well planned, as they must necessarily be in buildings on the barrack plan, they do not strike one as being briskly administered, and there are many signs that the United States Government would be well advised to take steps to introduce new blood and new methods in many directions.

We believe it would add greatly to the medical efficiency if the Marine Hospital service were to include the appointment of a Surgeon or Surgeons to act as a consulting staff, to pay at any rate weekly visits to each of these hospitals, and to introduce modern methods of treatment to a far greater extent than prevails at the present time.

So far as site is concerned this hospital may be regarded as ideal, but in many other respects it might be greatly improved, both in the interest of the patients and of the staff.

There seemed to us, from what little we saw of the place, to be too much delay in the treatment pursued, and the original appliances at the disposal of the medical staff were undoubtedly deficient, in one case—an ordinary Windsor chair being used to support the leg of a patient, no other means being apparently available.

The City and County Hospital is a large hospital with accommodation for about 400 patients. It was erected several years ago, and has been added to from time to time, and the city would be well advised to pull the pavilions down and erect in their place a modern hospital, replete with every sanitary adjunct which science can suggest.

The wards and corridors were anything but clean, and many of the baths, W.C.'s, and other places, were old and out of date, or dirty in appearance, whilst the whole institution bore the aspect of a place where workhouse rather than hospital methods were pursued. Personally, we should be very sorry to be a patient in this hospital at the present time, for, not only were many of the arrangements inadequate and uninviting, but the nursing, until the last two months, seems to have been wholly neglected, the mere appearance of the male nurses as we saw them in their mess room was such as to make us thankful that in British hospitals such a staff could nowhere be found.

Recently, the Board of Health have had the wisdom to apply to Philadelphia for two ladies to take charge of the nursing, with a view, we presume, to put it in a state of efficiency by the establishment of a nurse-training school in addition to trained nurses. The task of these ladies will be herculean, and, unless they are supported, we should counsel them to retire from a position which, under such circumstances, will be impossible. We gather, though we may be wrong in our impression, that the authorities do not regard with favour many of the necessary changes which these ladies have recommended.

Unfortunately, the hospital is largely the sport of political parties, and, so long as this state of things continues, so long must we expect to find inefficiency and discomfort to prevail throughout the entire establishment.

The hospital itself is situated outside the town. It is very difficult and most unfair to compel the poor who need the succour which the hospital affords to have to go so great a distance in search of the medical aid which their cases require.

There are few more beautiful or striking cities than the City of San Francisco. Everywhere there is an appearance of prosperity and wealth. We were told that it is the boast of its inhabitants that they number amongst them, at least, two hundred millionaires, and yet we understood that the chief hospital of the place is sadly stinted in regard to its supplies. It must be due to a want of thought, rather than to a want of heart, that not one of all the two hundred millionaires has as yet been led to take up this question of the adequate provision and proper supply of means for the relief of sickness and suffering within the city. Why not follow the example of Eastern merchants and of European millionaires who, to a large extent, build and endow hospitals on the just ground that there is not a single purpose to which they can more usefully or properly devote some of the wealth of which they are but the trustees for their lifetime.

It is all very well to make a great fortune in a few years, and to spend a certain portion of it in beautifying the city in which a man resides, or in erecting and laying out gardens and building summer residences, embellished by statues, flowers, shrubberies, and heights. This, no doubt, may be done to the honour and glory of God, but it is equally certain that it is done to the honour and glory of man, as any one who is acquainted with the city of San Francisco will be most painfully aware.

San Francisco is blessed with a Press which is ably directed, and, although that Press is largely political, we yet believe that the necessities of the suffering poor of the city and the neglectful conditions which, at present, characterise the arrangements for their accommodation have only to be brought prominently to the notice of the citizens to stir up one or more of the hundred millionaires and other philanthropists of this great city to prompt action. This will ensure that the condition of the halt and lame shall no longer be disregarded, and that the metropolis of the West shall be speedily and adequately provided with magnificent buildings, and a trained and capable staff to care for those of the citizens who have not been blessed with wealth, and who may have been struck down with sickness in the battle of life.

Until some such steps are taken, despite the splendour of this city, the beauty of its surroundings, the importance of its trade, and the wealth of its citizens, San Francisco must be regarded by the intelligent people of a free country as far behind the age in regard both to philanthropic enterprise, and the discharge of the duties which are incumbent upon all Christians, whatever may be their religious differences. In any case, no stranger who is adequately informed can visit San Francisco at the present time without a feeling of sorrow and regret, that here, under present conditions, the lot of the sick and needy is anything but enviable.

